# Reducing soil and leaf shadow interference in UAV imagery for cotton nitrogen monitoring

**DOI:** 10.3389/fpls.2024.1380306

**Published:** 2024-08-16

**Authors:** Caixia Yin, Zhenyang Wang, Xin Lv, Shizhe Qin, Lulu Ma, Ze Zhang, Qiuxiang Tang

**Affiliations:** ^1^ College of Agriculture, Xinjiang Agricultural University, Urumqi, China; ^2^ Key Laboratory of Oasis Eco-Agriculture, Xinjiang Production and Construction Corps, Shihezi University, Shihezi, China

**Keywords:** UAV, image pixels, vegetation index, leaf nitrogen content, hyperspectral

## Abstract

**Introduction:**

Individual leaves in the image are partly veiled by other leaves, which create shadows on another leaf. To eliminate the interference of soil and leaf shadows on cotton spectra and create reliable monitoring of cotton nitrogen content, one classification method to unmanned aerial vehicle (UAV) image pixels is proposed.

**Methods:**

In this work, green light (550 nm) is divided into 10 levels to limit soil and leaf shadows (LS) on cotton spectrum. How many shadow has an influence on cotton spectra may be determined by the strong correlation between the vegetation index (VI) and leaf nitrogen content (LNC). Several machine learning methods were utilized to predict LNC using less disturbed VI. R-Square (*R* ^2^), root mean square error (RMSE), and mean absolute error (MAE) were used to evaluate the performance of the model.

**Results:**

(i) after the spectrum were preprocessed by gaussian filter (GF), SG smooth (SG), and combination of GF and SG (GF&SG), the significant relationship between VI and LNC was greatly improved, so the Standard deviation of datasets was also decreased greatly; (ii) the image pixels were classified twice sequentially. Following the first classification, the influence of soil on vegetation index (VI) decreased. Following secondary classification, the influence of soil and LS to VI can be minimized. The relationship between the VI and LNC had improved significantly; (iii) After classifying the image pixels, the VI of 2-3, 2-4, and 2-5 have a stronger relationship with LNC accordingly. Correlation coefficients (*r*) can reach to 0.5. That optimizes monitoring performance when combined with GF&SG to predict LNC, support vector machine regression (SVMR) has the better performance, *R* ^2^, RMSE, and MAE up to 0.86, 1.01, and 0.71, respectively. The UAV image classification technique in this study can minimize the negative effects of soil and LS on cotton spectrum, allowing for efficient and timely predict LNC.

## 1 Introduction

Nitrogen is an essential element for crop growth and development, and its abundance and deficiency have great influence on cotton yield and quality (Snider et al., 2021). Traditional nitrogen monitoring methods require human and material resources during sampling, determination, and data analysis, among other processes, and appear to be not commonly applied (Bacsa et al., 2019). The rapid development of spectral technology, particularly rapid non-destructive and spectrum integrated monitoring technology, has balanced the limitations of traditional nitrogen monitoring methods (Johnson et al., 2016).

Precision agriculture is vital for cotton nitrogen monitoring by gathering field data for precision management and decision making in big fields. Spectral technology has become an important direction and research hotspot in precision agriculture. Hyperspectral remote sensing extracts spectroscopic data from a target using electromagnetic spectra with extremely narrow wavelengths, generating a continuous and full spectral curve with spectral information perfectly reflecting the intrinsic features of the target (Govender et al., 2008). At present, remote sensing technology is divided into aerospace, aviation, low-altitude remote sensing, and near-ground remote sensing. Low-altitude remote sensing is the collection of data via unmanned aerial vehicle (UAV) photography (Sun et al., 2017). Compared with near-ground remote sensing, UAV remote sensing has higher temporal and spatial resolution (Zheng et al., 2018), tend to be highly flexible and relatively inexpensive, and is suitable for large-scale monitoring (Delavarpour et al., 2021), whereas remote sensing from aerospace and aviation has a time and space lag with crops (McConkey et al., 2004; Yang et al., 2014), in addition to several limitations due to cloud coverage and high costs, making UAV technology a complementary to near-ground and satellite technology (Manfreda et al., 2018). In agriculture, much of the work is done using UAV, including growth assessment (Tao et al., 2020; Gong et al., 2021) and nutrition assessment, among others (Kerkech et al., 2020; Das et al., 2022).

Academic research on nutrition assessment has been conducted on a variety of crops, such as winter wheat (Sun et al., 2018; Song et al., 2022), rice (Bacsa et al., 2019), corn (Xu et al., 2021), cotton (Chu et al., 2016; Volkova et al., 2018), and sorghum (Shafian et al., 2018). However, there are still significant barriers to agricultural nutrition monitoring, such as the complicated growing environment of field crops, which results in UAV images with a huge quantity of soil and leaf shadow, among others. According to Liu et al. (2020), the key elements influencing the accuracy of monitoring the aforementioned agricultural information include the specific vegetative growth stages, the vegetation cover, the crop canopy structure, and the crop types. This may be the result of non-static cotton leaf shadow or soil. Cotton has a dwarf form and more leaves. The lower leaves in the binary images are partially or entirely shaded by canopy leaves, casting shadows on another leaf. While the reflectance of shadows is likewise relatively lower, these areas are either explicitly directly deleted or ignored in target recognition, leading to the acquisition of vegetation spectra lower than the actual spectral reflectance (Tong et al., 2017; Liu et al., 2020). To increase the inversion performance, some researchers have used the canny edge detection technique to eliminate the soil background and leaf shadow (Zhang et al., 2018). Caturegli et al. (2015) claimed that the magnitude of the normalized difference vegetation index (NDVI) is vulnerable to the surrounding environment. To minimize as much as possible the influence of soil and leaf shadows on the cotton spectrum, a significant amount of research has been conducted by numerous experts on ways to lessen the interference of soil and leaf shadows on the spectrum, which is the most valuable information for precision farming. Three points are summarized below. Firstly, specific spectral pretreatment techniques can successfully minimize the disturbance of soil, such as the first-order derivative and Savitzky–Golay (SG) smoothing (Zhang et al., 2022; Yin et al., 2022). Secondly, the vegetation index (VI) is a product of the enhanced remote sensing image that can efficiently emphasize spectral features while reducing redundant information, which is more stable and dependable than spectral reflectance (Feng et al., 2014; Yao et al., 2014). For example, Chen et al. (2010) proposed that the double-peaked canopy nitrogen index (DCNI) could mitigate the effect of canopy structure. Stroppiana et al. (2009) claimed that the optimal normalized difference index (NDIopt) based on the blue/green regions has a smaller impact on the rice leaf area index (LAI) and canopy structure than the NDVI and is more sensitive to changes in the plant N concentration. Zhu et al. (2007) demonstrated that the link between various VIs and N changed with crop type and crop phenology. As a result, the VI may be more stable for target detection than spectral reflection information. Thirdly, strategies to reduce the interference of soil and shadow have been developed by many scholars. Woebbecke et al. (1995) evaluated the capacity of various color indices to differentiate the vegetation from the background and hypothesized that the excess green (ExG) vegetation index may offer a near-binary intensity image to highlight a plant area of interest. M-Desa et al. (2022) performed picture shadow and non-shadow discrimination using color characteristics and supervised classification. There are numerous studies on the shadow rejection of buildings in UAV images; however, for agricultural images, these studies have all focused on trees with simple canopy structures (Prado Osco et al., 2019), such as apples and oranges, where shadows are easy to reject (Khekade and Bhoyar, 2015). Therefore, leaf shadows are difficult to remove completely. With advancement of the phenological stage of cotton, cotton leaves will gradually expand and the soil in the captured image could be covered. Therefore, the impact of soil on cotton field nutrient monitoring can be easily removed than the leaf shadow. Thus, rejecting cotton leaf shadowing is sophisticated compared with cotton nitrogen monitoring. Woebbecke et al. (1995) proposed that the color difference coordinates of green were significantly higher than those of other colors and used red–green–blue (RGB) primary colors to determine a color index that best distinguishes between the various color images of plant materials, weeds, soil, residues, and background light conditions. Guyer et al. (1986) discovered that the intensity of the pixels in the plant images was higher than that of the soil pixels by shining visible light on the plants.

Undeniably, in addition to these factors that might affect the cotton leaf spectra, modeling methods can also directly contribute to the precision of forecasts. Miphokasap et al. (2012) provided proof that, in comparison to narrow-band multiple linear regression (MLR)-based model applications, the model model developed by SMLR demonstrated a higher correlation coefficient with nitrogen content than the model based on narrow vegetation indices. In recent decades, numerous nonlinear and nonparametric methods that go beyond linear regression and linear transformation have been developed. These methods are also known as machine learning regression algorithms (Verrelst et al., 2015). Therefore, this study classified image pixels to eliminate redundant information and to reduce the impact of leaf shading and soil on the drone spectra. Machine learning methods were used to improve the accuracy of the large-scale monitoring of cotton leaf nitrogen content (LNC) in order to provide guidance for the rational application of nitrogen fertilizer.

## 2 Materials and methods

### 2.1 Experimental area

**Figure 1:**
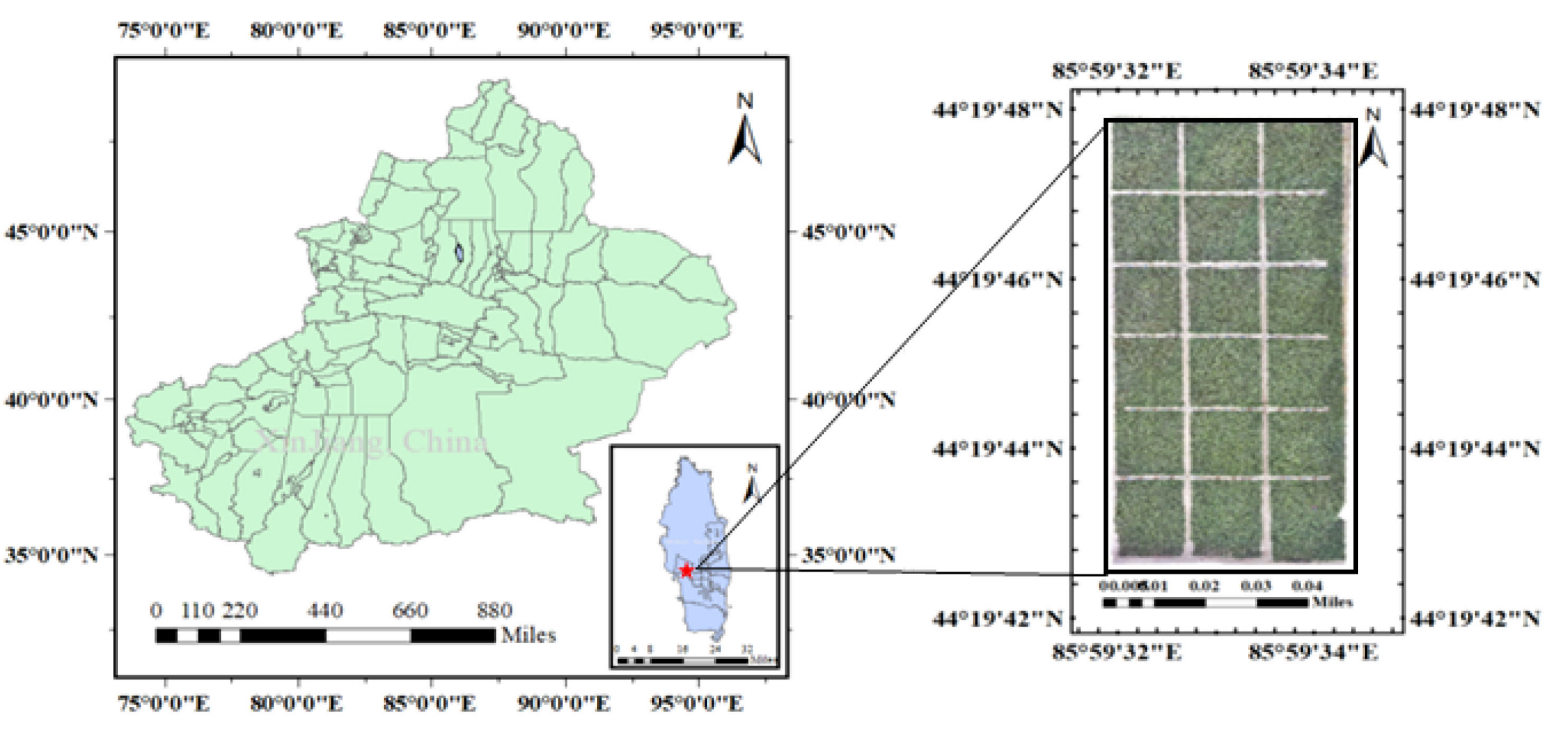
Experimental area.

This experiment was carried out in July of 2020 at the experimental station of Shihezi University (44°19′53.83″ N, 85°59.65″ E). **Figure 1** depicts the map of the experimental area. Various amounts of N fertilizer were randomly applied to each plot, resulting in a total of 6 N fertilizer gradients and three repetitions. There were 18 pilot zones.

The application of six N fertilizers was intended to enhance the presence of numerous N fertilizers. The nitrogen fertilizer gradients were 0, 120, 240, 360, and 480 kg ha^−1^ to ensure the stability of the model estimates, which were correspondingly indicated as N0, N120, N240, N360, and N480. Cotton was the first plant to be cultivated at the experimental site. Prior to planting, 30% N fertilizer was applied to the cotton. The second half of April was used to grow cotton. Of the N fertilizer, 70% was sprayed on June 22, July 9, July 18, August 5, and August 15 at respective rates of 10%, 10%, 20%, 20%, and 10%. The water was irrigated on June 13, June 23, July 14, July 25, August 5, and August 16 according to the combined local standard drip irrigation.

### 2.2 Data collection

#### 2.2.1 Hyperspectral images using an unmanned aerial vehicle

The UAV images were captured at 100 m above the cotton canopy on July 16, 2020. Clear weather and the absence of wind and clouds are essential for capturing the images. The flight was scheduled to depart between 1200 and 1400 hours local time.

When the UAV was flying at 100 m altitude, the ground spatial resolution was 0.03 m. The UAV is an aircraft in the domestically DJI M600 series (**Supplementary Figure 1**). The sensor used was Nano-Hyperspec (**Supplementary Figure 2**). The sensor has a GPS integrated inertial measurement unit (IMU). The GPS and IMU signals were used for orthorectification. The parameters are listed in **Table 1**.

**Table 1:** Main parameters of the nano-imaging spectrometer.

Parameter	Value
Name	Nano-Hyperspec
Spectral band range (nm)	400–1,000
Spectral interval (nm)	270
Spectral resolution (nm)	6
Sampling interval (nm)	2.2
Space channel	640
Aircraft quality (kg)	0.6

#### 2.2.2 Cotton leaf nitrogen content

For this study, cotton plants were destructively sampled during their bud phase. The chopped leaves were placed in ice trays and returned to the laboratory for LNC measurements. The leaves were oven-dried to constant weight at 80°C and then crushed using a crusher. The cotton LNC was determined through micro-Kjeldahl analysis (Tian et al., 2014).

### 2.3 Data processing methods

#### 2.3.1 UAV image pre-processing

**Figure 2:**
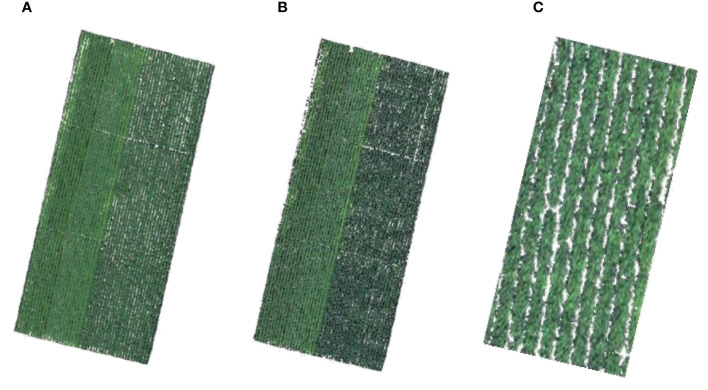
**(A)** Red–green–blue (RGB) map of cotton in the experiment area. **(B)** True color map after removing the soil visible to the naked eye, with *white* displaying the soil and the background and *green* representing the cotton. **(C)** Enlargement of the cotton in **(B)**.

The acquired UAV images were pre-processed with the instrument’s own software, “Spectral View,” which also included atmospheric and orthorectification adjustments. *ENVI* 5.3 was used to complete the image mosaic and radiometric calibration procedures. Radiometric calibration is the conversion of the normalized difference (ND) values into spectral reflectance. The reflectance of UAV at the same physical position as the LNC sample point was extracted. Before extraction, the soils that can be seen with the naked eye were excluded via unsupervised classification, leaving just the vegetation for further investigation (**Figure 2**).

#### 2.3.2 Classification method of the image pixels

**Figure 3:**
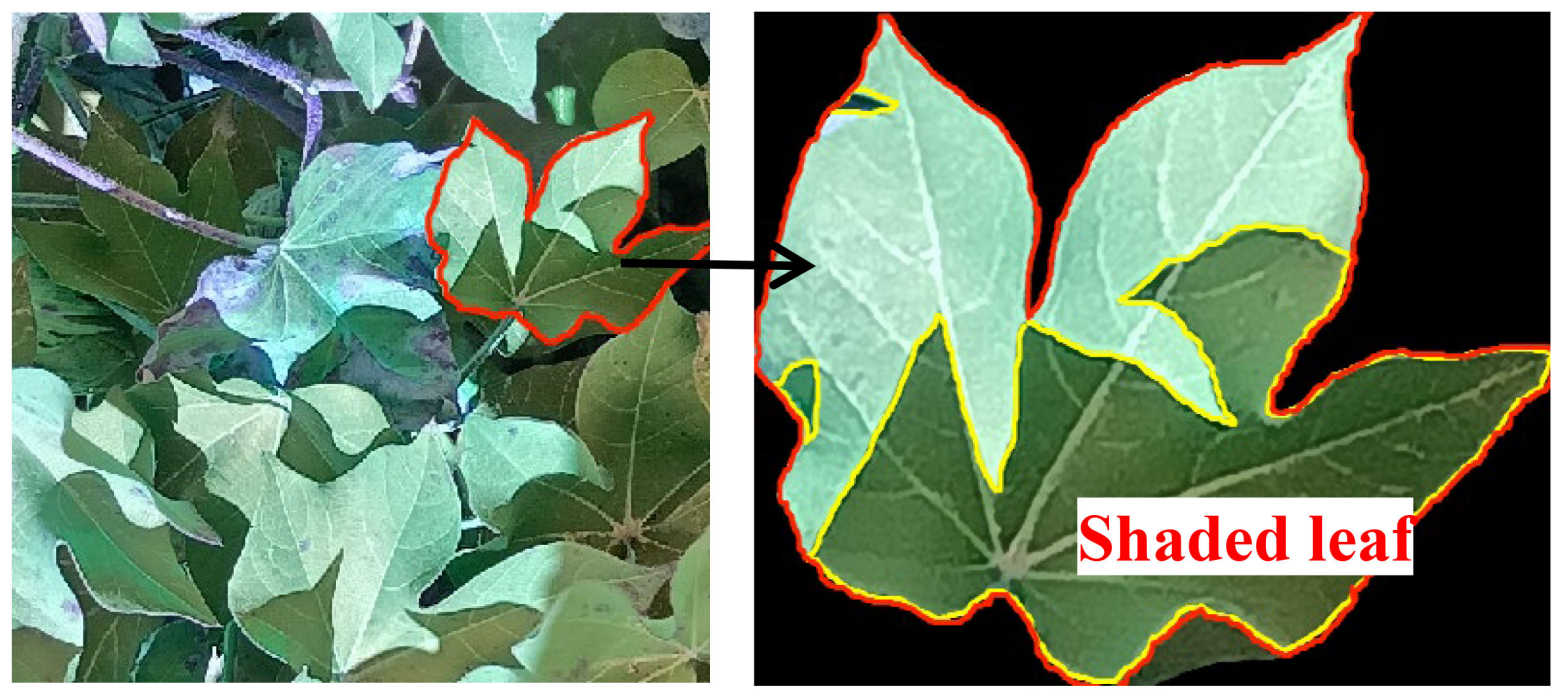
Non-shaded leaf and shaded leaf.

**Figure 4:**
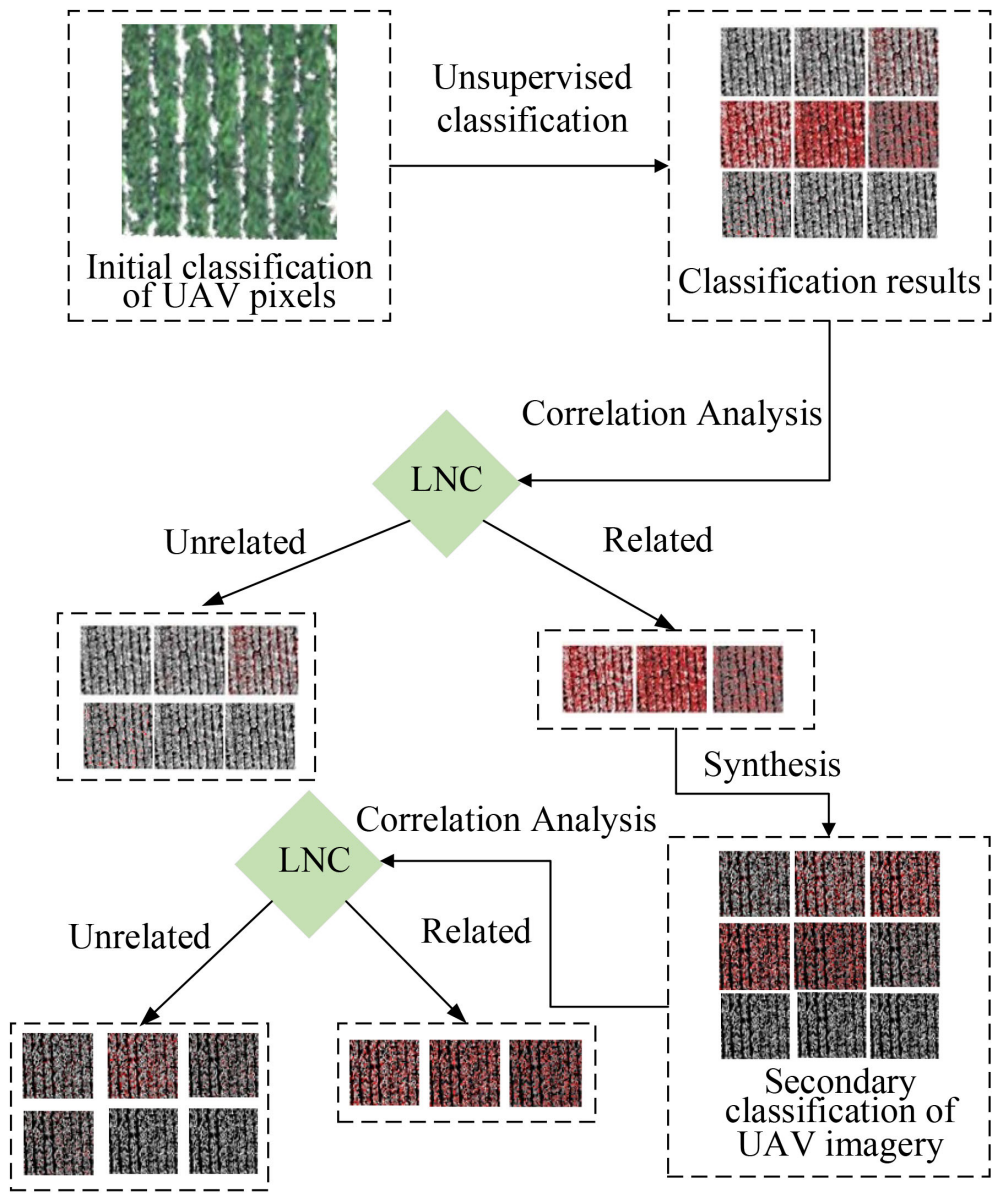
Classification flowchart.

As the image is susceptible to shadow interference (**Figure 3**), in this study, green light was extracted for image pixel classification. Refer to **Figure 4** for the classification process. Focusing on the green light value, all image pixels were classified into 10 categories.

In this study, two classifications were performed, and the 10 categories for the first classification were labeled as follows: 1-0, 1-1, 1-2, 1-3, 1-4, 1-5, 1-6, 1-7, 1-8, and 1-9. After determining that 1-4, 1-5, and 1-6 adequately represented the cotton canopy, a second classification was performed in conjunction with 1-4 and 1-5, which were labeled as 2-0, 2-1, 2-2, 2-3, 2-4, 2-5, 2-6, 2-7, 2-8, and 2-9. Following classification, the extracted hyperspectral reflectance corresponding to each group was used to generate the VI. The results are listed in **Table 2**. In this investigation, 20 spectral indices with the highest association with N were chosen from the available literature for content analysis and model development. Refer to **Table 2** for the index results.

**Table 2:** Vegetation indices used in this study.

No.	Spectral index	Calculation formula	Reference
1	Zarco-Tejada–Miller index	ZMI = *R*_750_/*R*_710_	Zarco-Tejada et al. (2001)
2	Vogelmann red edge index 1	VOG1 = *R*_740_/*R*_720_	Vogelmann et al. (1993)
3	Green–red vegetation index	GRVI = *R*_800_/*R*_550_	Gitelson et al. (2005)
4	Resistant vegetation index	RVI = *R*_800_/*R*_700_	Merzlyaket al. (2003)
5	Red/green ratio	R/G = *R*_Red_/*R*_green_	Sims and Gamon (2002)
6	Modified anthocyanin content index	MACI = *R*_NIR_/*R*_green_	Gitelson et al. (2010)
7	Red edge chlorophyll index	RECI = (*R*_750_/*R*_550_) − 1	Gitelson et al. (2010)
8	Gitelson and Merzlyak index	GMI = (*R*_750_/*R*_720_) − 1	
9	Green normalized difference vegetation index	GNDVI = (*R*_780_ − _550_)/(*R*_780_ + *R*_550_)	Leprieur et al. (2000)
10	Normalized difference vegetation index	NDVI = (*R*_810_ − *R*_560_)/(*R*_810_ + *R*_560_)	Aparicio et al. (2000)
11	Infrared percentage vegetation index	IPVI = *R*_800_/(*R*_800_ + *R*_670_)	Crippen (1990)
12	Meris terrestrial chlorophyll index	MTCI = (*R*_754_ − *R*_709_)/(*R*_709_ − *R*_681_)	Dash and Curran (2007)
13	Structure insensitive pigment index	SIPI = (*R*_800_ − *R*_450_)/(*R*_800_ + *R*_450_)	Penuelas et al. (1995)
14	Modified simple ratio 705	mSR_705_ = (*R*_750_ − *R*_445_)/(*R*_705_ + *R*_445_)	Sims and Gamon, 2002
15	Normalized difference vegetation index 801	NDVI_801_ = (*R*_801_ − *R*_550_)/(*R*_801_ + *R*_550_)	Gitelson et al. (2005)
16	Red edge normalized difference vegetation index	RENDVI = (*R*_750_ − *R*_705_)/(*R*_750_ + *R*_705_)	Gitelson and Merzlyak (1994)
17	Modified red edge normalized difference vegetation index	mND_705_ = (*R*_750_ − *R*_705_)/(*R*_750_ + *R*_705_ − 2*R*_445_)	Sims and Gamon (2002)
18	Normalized phaeophytinization index	NPQI = (*R*_415_ − *R*_435_)/(*R*_415_ + *R*_435_)	Barnes et al. (2000)
19	Hyperspectral normalized difference vegetation index	HNDVI=(*R*_827_ − *R*_668_)/(*R*_827_ + *R*_668_)	Oppelt and Mauser (2004)
20	Soil-adjusted vegetation index	SAVI = 1.5(*R*_800_ − *R*_670_)/(*R*_800_ − *R*_670_ + 0.5)	Rondeaux et al. (1996)

R_415_, R_435_, R_445_, R_450_, R_550_, R_668_, R_670_, R_681_, R_700_, R_705_, R_709_, R_710_, R_720_, R_740_, R_754_, R_780_, R_800_, R_810_, R_801_, and R_827_, represent the spectral reflectance values of the cotton canopy at wavelengths of 415, 435, 445, 450, 550, 668, 670, 681, 700, 705, 709, 710, 720, 740, 75, 754, 780, 800, 810, 801, and 827 nm, respectively. R_Red,_ R_green_, and R_NIR_ are the red, green, and near-infrared reflectance values, respectively.

#### 2.3.3 Extraction of spectral reflectance

The region of interest (ROI) was divided on the corresponding position of the UAV image, and the average spectral information of ROI was used as the spectral value of this sampling point. There were two types of ROI. The first ROI was for acquiring 900 (30 × 30) pixels around the sampling point, which was 0.9 m × 0.9 m and covered an entire row of cotton. **Supplementary Figure 3** shows a schematic representation. The second ROI was for obtaining the mean values of all spectra within 900 pixels at each sampling point after identification of the image pixels.

#### 2.3.4 Spectral pre-processing

In order to minimize the influence of noise and soil on the collected spectra, three pretreatments were applied to the original spectrum (OS) reflectance. The OS was without pre-processing.

Gaussian filter (GF) is a linear smoothing filter used to remove Gaussian noise in image processing. Application of a GF to UAV spectral reflectance generates the appropriate VI for modeling. Savitzky and Golay invented SG smoothing, also called convolutional smoothing. The signal form and width are preserved while the noise is removed. After SG smoothing of the UAV spectral reflectance, the appropriate VI is produced for modeling. Gaussian and SG (GF&SG) means that the Gaussian-processed spectrum was further smoothed with SG.

#### 2.3.5 Methods for predicting LNC

Based on the Pearson’s coefficient of correlation approach to define the VI substantially correlated with the nitrogen content as the independent variable of the model, partial least squares regression (PLSR), MLR, principal component regression (PCR), and support vector machine regression (SVMR) were used to develop cotton nitrogen content estimation models. *R* ^2^, the root mean square error (RMSE), and the mean absolute error (MAE) were used to evaluate the performance of the model (Yin et al., 2022).

## 3 Results

### 3.1 De-interference effect of spectral pretreatment

**Figure 5:**
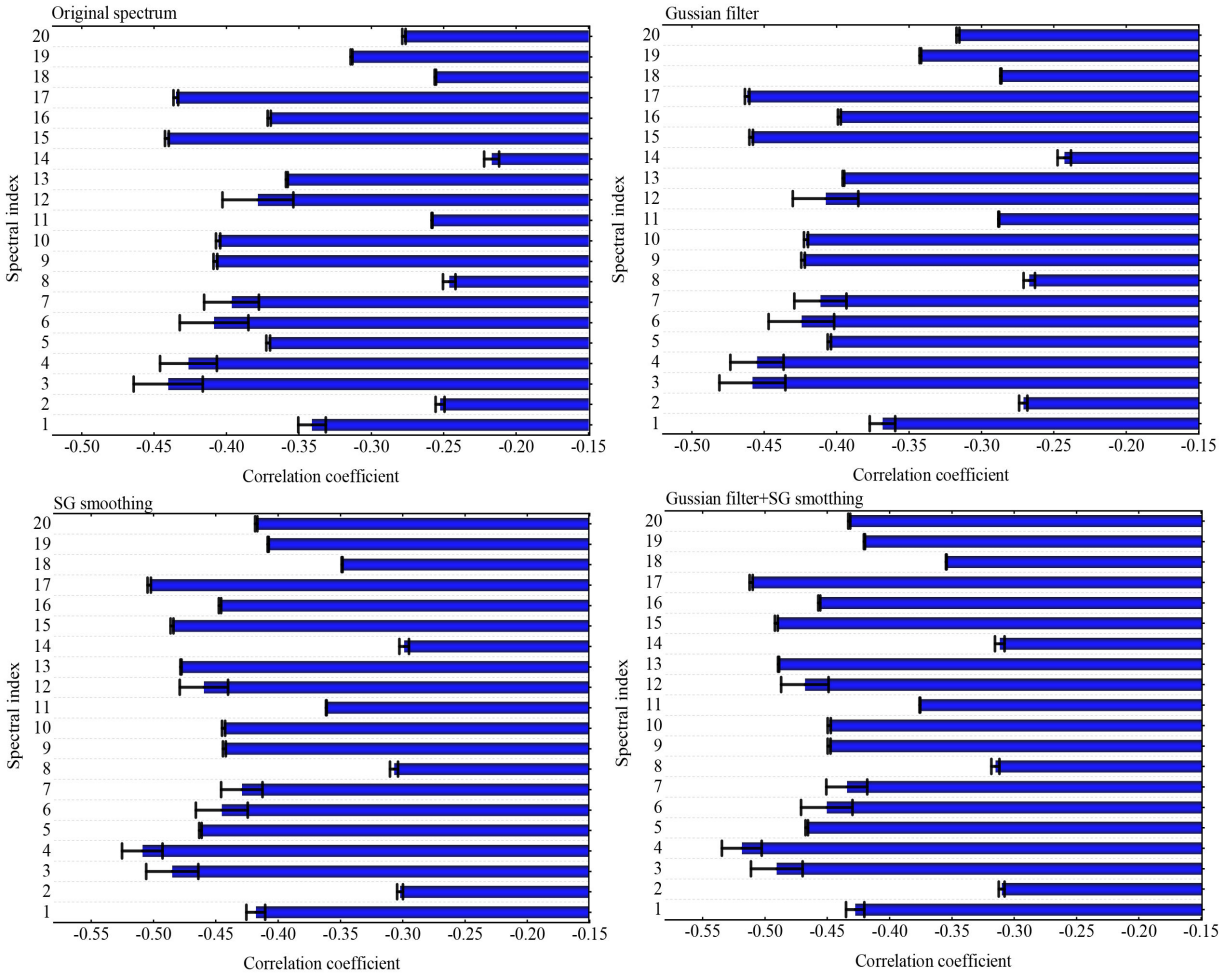
Correlation coefficients between the leaf nitrogen content (LNC) and the vegetation index (VI) with pretreatments.

In this paper, reflectance was obtained from the UAV hyperspectral images and pre-processed using GF, SG, and GF&SG. After pre-processing of the spectra, the indices were calculated and compared (**Figure 5**). The results revealed that all 20 VIs demonstrated significant correlations with the cotton LNC. The correlation coefficients between the pretreated spectral indices calculated using GF, SG, and GF&SG and the LNC of cotton were successively increased. There were seven indices with correlation coefficients above 0.4 between the OS and the cotton LNC: the green–red vegetation index (GRVI), the resistant vegetation index (RVI), the modified anthocyanin content index (MACI), the NDVI (780, 550) (810, 660), the green normalized difference vegetation index (GNDVI), and the modified red edge normalized difference vegetation index (mND_705_), with correlation coefficients of −0.44**, −0.43**, −0.41**, −0.41**, −0.44**, and −0.43**, respectively. The correlation coefficients of these seven indices with the cotton LNC were improved by 0.02, 0.03, 0.02, 0.02, 0.02, 0.02, and 0.03, respectively, after GF pretreatment. After pretreatment with SG, these indices were improved by 0.04, 0.08, 0.04, 0.04, 0.04, 0.04, and 0.07, respectively. After pretreatment with GF&SG, the correlation coefficients improved by 0.05, 0.09, 0.04, 0.04, 0.04, 0.05, and 0.08, respectively. Therefore, pretreatment with GF&SG significantly improved the association between VI and cotton LNC.

**Figure 6:**
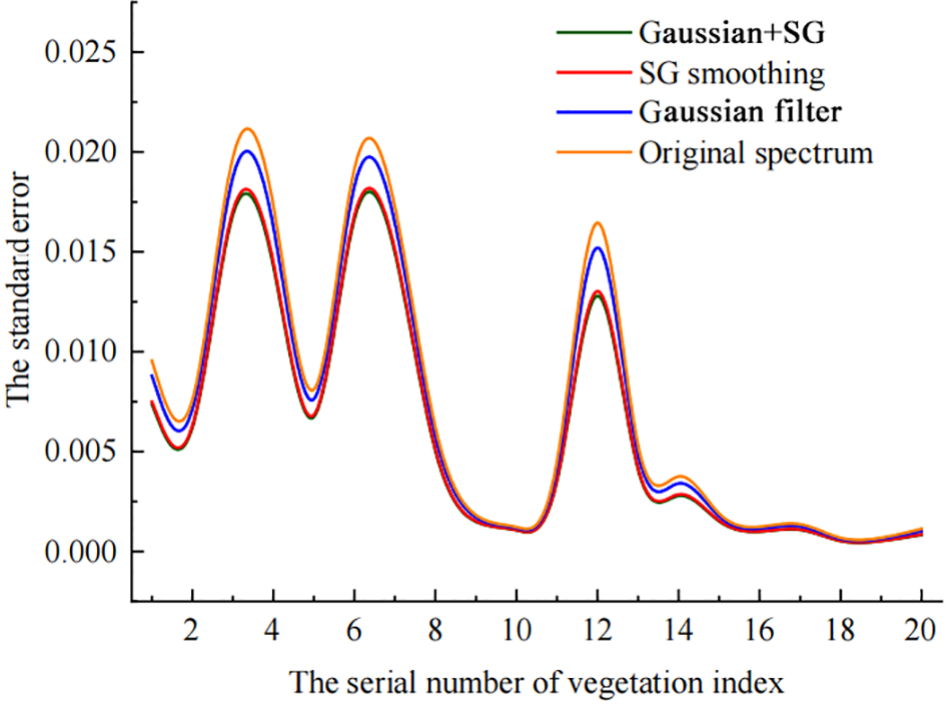
Standard errors of the vegetation indices with pretreatments.

This study analyzed the changes in sample errors following pretreatment of 108 samples (**Figure 6**). A number of spectrum samples had higher standard errors, including GRVI, RVI, MACI, the Gitelson and Merzlyak index (GMI), and the Meris terrestrial chlorophyll index (MTCI), which reached roughly 0.02, whereas the other indices had less change before and after pretreatment. These five indices with substantial standard error variations are susceptible to environmental conditions such as soil, while the others are not. After several pretreatments, the standard errors were dramatically reduced, indicating that pretreatment can eliminate the effect of soil on the index.

**Table 3** displays the results of the significance tests of the derived indices for the OS and after three pretreatments. After pretreatment, the VIs were significantly different from the OS. No substantial difference exists between the indices for GF and SG, indicating that they have similar functions. The indices computed after GF&SG were significantly different from those calculated with the single treatment, indicating that the overlap of the GF&SG can significantly increase the single pretreatment effect in reducing the interference of soil.

**Table 3:** Significance test of the vegetation indices before and after pretreatment.

Spectral index	OS	GF	SG	GF-SG	Spectral index	OS	GF	SG	GF-SG
ZMI	0.10 b	3.05 a	3.05 a	3.05 a	IPVI	0.51 c	0.89 b	0.89 b	1.23 a
VOG1	1.01 c	1.71 b	1.71 b	2.84 a	MTCI	0.21 c	3.85 a	3.82 a	1.24 b
GRVI	7.36 a	5.23 b	5.22 b	2.61 c	SIPI	0.65 c	0.75 b	0.75 b	1.16 a
RVI	0.10 c	5.10 a	5.09 a	2.43 b	mSR_705_	0.68 c	1.94 a	1.94 a	1.09 b
R/G	5.92 a	0.71 c	0.71 c	2.06 b	NDVI_801_	0.76 b	0.68 c	0.68 c	1.07 a
MACI	7.39 a	5.17 b	5.16 b	1.94 c	RENDVI	0.00 c	0.77 b	0.77 b	1.04 a
RECI	6.44 a	3.66 b	3.66 b	1.64 c	mND_705_	0.00 c	0.58 b	0.58 b	1.06 a
GMI	0.00 c	0.92 b	0.92 b	1.56 a	NPQI	0.12 c	0.07 b	0.07 b	1.02 a
GNDVI	0.76 b	0.67 c	0.67 c	1.41 a	HNDVI	0.00 c	0.78 b	0.78 b	1.04 a
NDVI	0.68 c	0.68 c	0.76 b	1.35 a	SAVI	0.12 c	0.76 b	0.76 b	1.04 a

Different lowercase letters in the same column indicate significant differences in the vegetation index before and after pretreatment (p < 0.05). See **Table 2** for the definitions of the different indices.

OS, original spectra; GF, Gaussian filter; SG, Savitzky–Golay smoothing; GE-SG, Gaussian filtering + Savitzky–Golay smoothing.

### 3.2 Pixel classification

The results of the classification of the image pixels into cotton and others are displayed in **Table 4**. Cotton comprised a total of 1,273,202 image pixels, or 65.74% of the total image pixels.

**Table 4:** Statistics on image pixel information.

	Total no. of pixels	Proportion (%)
Total no. of pixels	1,936,498	100
Total cotton image pixels	1,273,202	65.75
Total pixels of the other images	663,296	34.25

**Figure 7:**
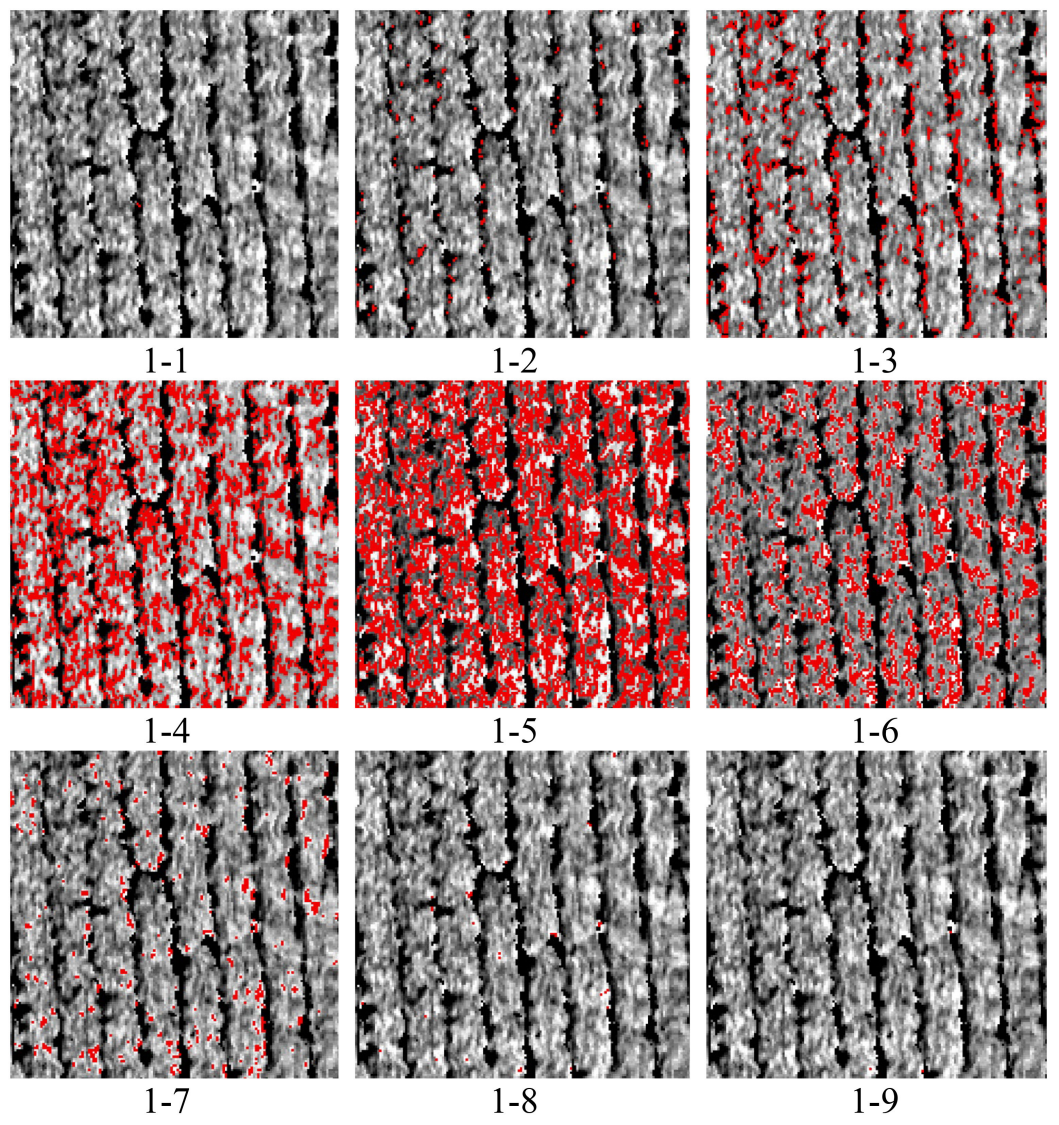
Classification results.

Consequently, this study focused on green light and separated it into 10 classes proportional to its intensity. The spectral reflectance corresponding to each class was then determined and labeled as 1-0, 1-1, 1-2, 1-3, 1-4, 1-5, 1-6, 1-7, 1-8, and 1-9 (**Figure 7**). Viewed in conjunction with the classification results in **Figure 7**, among them, the result for 1-0 was the same as that for 1-1 and, considering the layout, was not included in the figure. It was apparent that 1-4, 1-5, and 1-6 were all at the edge position of the cotton and soil and that the soil can be effectively reduced. Moreover, 1-1, 1-7, 1-8, and 1-9 indicate that the image pixels accounted for very little information and that almost none was expressed.

**Figure 8:**
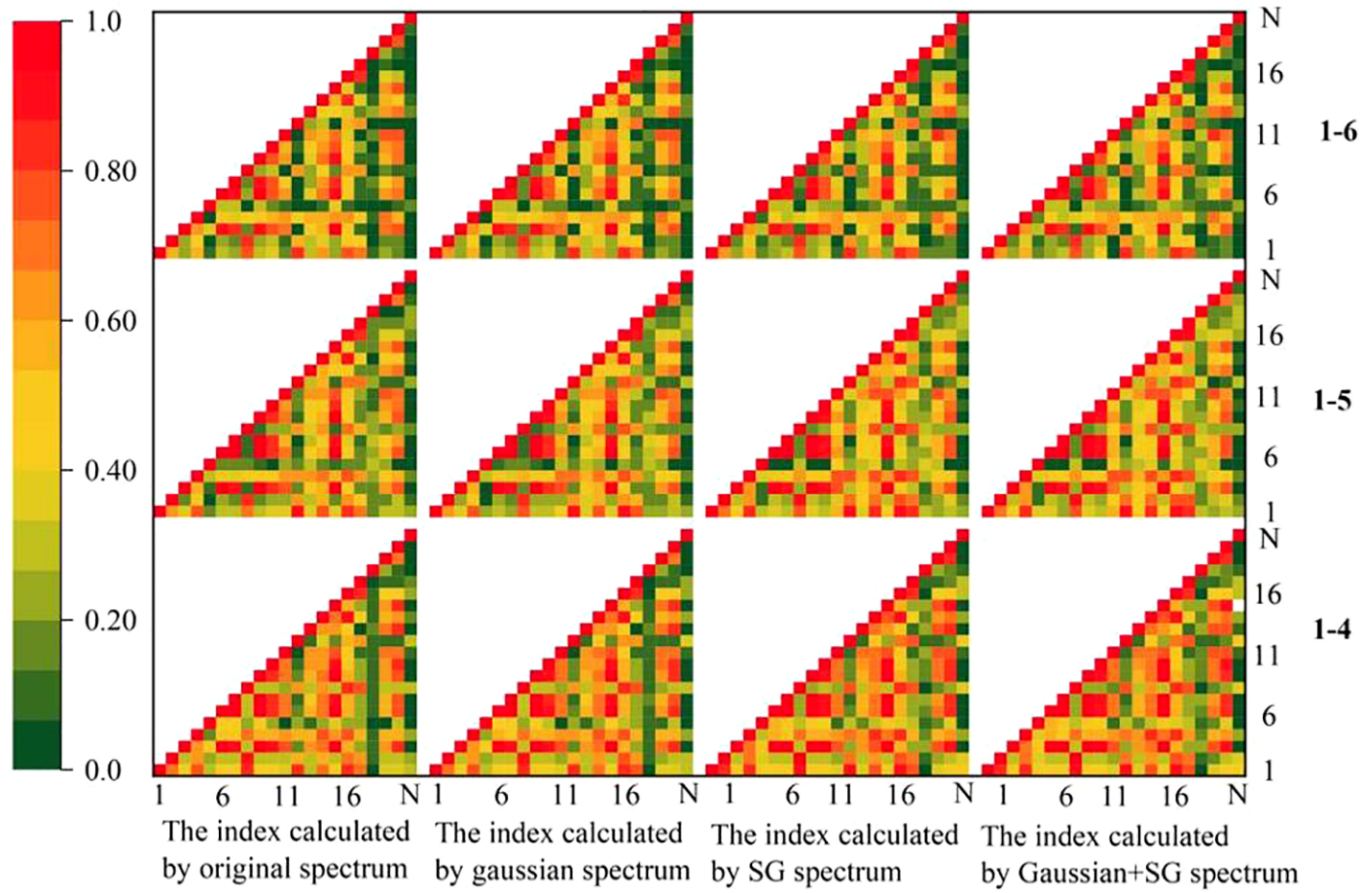
Correlation analysis of the vegetation indices. *SG*, Savitzky–Golay smoothing.

As a result, after classification, the spectral indices of 1-4, 1-5, and 1-6 were obtained for this investigation. The results revealed that the index values corresponding to each class after classification differed significantly. The correlation coefficients were calculated for each category of VIs. **Figure 8** shows that the indices were highly correlated with each other. In general, the normalized phaeophytinization index (NPQI) of 1-4 and 1-6 had low correlation coefficients with the other indices. None of them exceeded 0.4, while the maximum correlation coefficient between the NPQI of 1-5 and the other indices was close to 0.6. This is because NPQI is a normalized index with two bands, 415 and 435 nm, while all other indices had reflectance in the near-infrared region (NIR) [at around 780 nm, except for the red/green ratio (R/G)]. The correlation coefficients among the other indices were consequently high. The correlation coefficients of R/G determined in 1-5 and 1-6 were similarly relatively negative with the other indices, as R/G is a ratio index derived from the reflectance of the red and green bands and therefore had a negative correlation with the other indices. Overall, the correlation coefficients between indices 1-4, 1-5, and 1-6 were high, but those among the individual indices were low. For instance, the correlation coefficients between the Zarco-Tejada–Miller index (ZMI) and Vogelmann red edge index 1 (VOG1) for 1-6 did not reach 0.4 with R/G, MACI, the red edge chlorophyll index (RECI), GNDVI, infrared percentage vegetation index (IPVI), structure insensitive pigment index (SIPI), NPQI, the hyperspectral normalized difference vegetation index (HNDVI), and the soil-adjusted vegetation index (SAVI), although the correlation coefficients of 1-4 and 1-5 all increased. This shows that the picture pixel classification method had a significant impact on the index.

**Figure 9:**
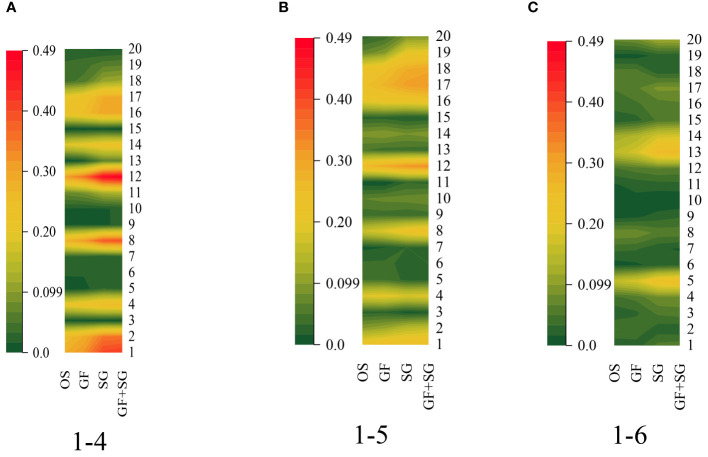
Relationship between the vegetation index (VI) and the leaf nitrogen content (LNC). **(A)** 1-4. **(B)** 1-5. **(C)** 1-6.

The coefficients of correlation were computed between the VI and the cotton LNC (**Figure 9**). The results indicated that the ZMI, VOG1, RVI, GMI, MTCI, the modified simple ratio 705 (mSR_705_), the red edge normalized difference vegetation index (RENDVI), and the modified red edge normalized difference vegetation index (mND_705_) of 1-4, ZMI, GMI, MTCI, RENDVI, mND_705_, NPQI, HNDVI exhibited a significant relationship with the cotton LNC, whereas the GRVI, R/G, MACI, RECI, GNDVI, NDVI, IPVI, SIPI, and SAVI were not substantially correlated with the cotton nitrogen content. Therefore, 1-6 was excluded from this study, and 1-4 was paired with 1-5 (C4&5) for reclassification.

### 3.3 Quadratic classification of images

**Figure 10:**
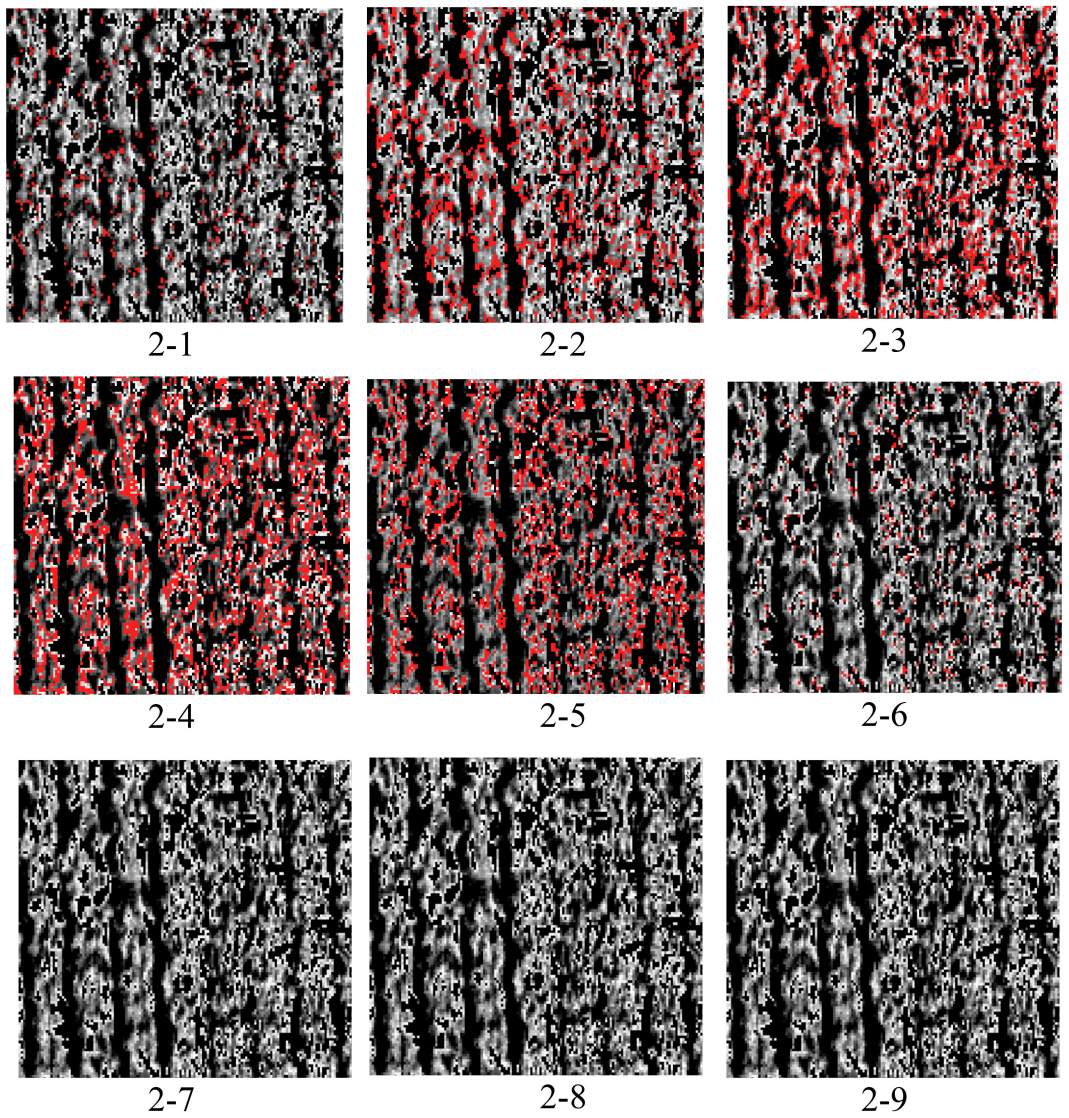
Reclassification results. For the classification outcomes for C4&5, the results are 2-3, 2-4, and 2-5. The correlation coefficients were calculated for each category of vegetation indices.

Observation of the second classification results (**Figure 10**) revealed that the result for 2-0 was the same as that for 2-1 and, considering the layout, was therefore not included in the figure. It was also found that 2-3 (category 4), 2-4 (category 5), and 2-5 (category 6) might have been more representative of cotton.

**Figure 11:**
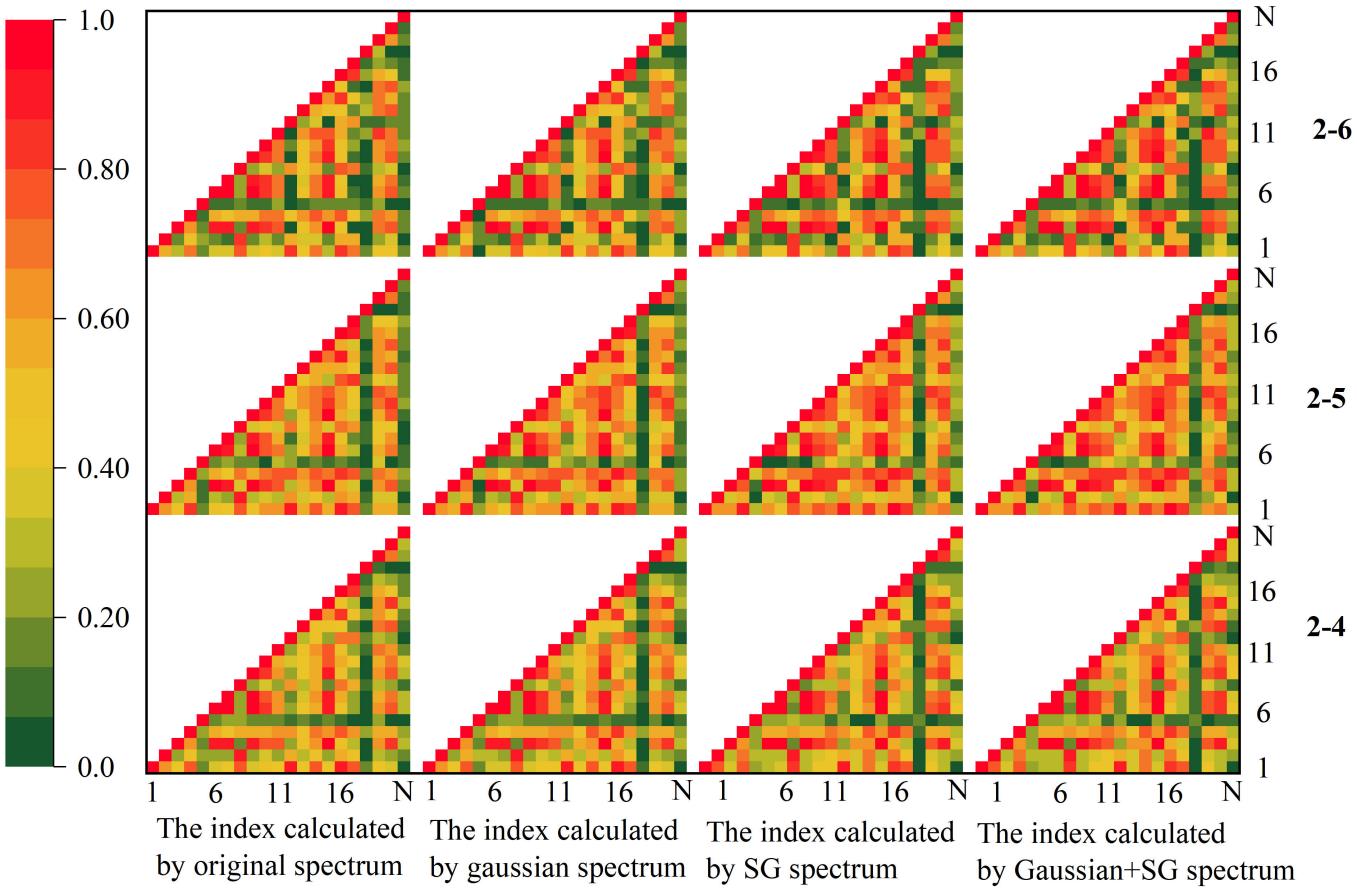
Correlation analysis on the vegetation indices classified as secondary. *SG*, Savitzky–Golay smoothing.

The correlation coefficients among the indices dropped since the initial categorization, as seen in **Figure 11**. Overall, the correlation coefficients between index 18 and index 5 and the other indices were modest and did not surpass 0.4, in general, for the same reason as that of the first categorization results. The other indicators showed strong relationships with each other, with the majority of the values being greater than 0.4. The correlation coefficients among a few particular individual indices were also lower; for instance, the correlation coefficients between MACI or RECI and mND_705_ did not exceed 0.6, and the correlation coefficients among the individual indices of 2-6 were lower than those of the other two categories. **Table 5** displays the results of the significance tests conducted on the indices. It can be inferred that the indices calculated by the three classes showed substantial discrepancies; however, VOGI, SIPI, NPQI, and HNDVI did not, indicating that these indices were more stable and less susceptible to external influences. The rest of the indices exhibited substantial variance, indicating that they were less stable than the four aforementioned indices.

**Table 5:** Significance test of the vegetation indices after the second classification.

Spectral index	2-3	2-4	2-5	Spectral index	2-3	2-4	2-5
ZMI	2.99 b	3.07 a	2.99 b	IPVI	0.88 a	0.88 a	0.84 b
VOG1	1.69 a	1.70 a	1.68 a	MTCI	3.72 ab	3.85 a	3.65 b
GRVI	4.96 a	4.94 ab	4.82 b	SIPI	0.75 a	0.75 a	0.75 a
RVI	4.99 b	5.13 a	5.04 ab	mSR_705_	1.89 b	1.94 a	1.93 ab
R/G	0.69 a	0.68 a	0.66 b	GNDVI	0.66 a	0.66 ab	0.65 b
MACI	4.89 a	4.83 ab	4.73 b	RENDVI	0.56 b	0.58 a	0.56 b
RECI	3.44 a	3.40 ab	3.31 b	mND_705_	0.77 a	0.77 a	0.76 b
GMI	0.89 ab	0.93 a	0.88 b	NPQI	0.07 a	0.07 a	0.07 a
GNDVI	0.66 a	0.66 ab	0.65 b	HNDVI	0.77 a	0.77 a	0.77 a
NDVI	0.66 a	0.66 ab	0.65 b	SAVI	0.74 b	0.76 a	0.76 a

Different lowercase letters in the same column indicate significant differences in the vegetation index before and after pretreatment (p < 0.05). See **Table 2** for the definitions of the different indices.

**Figure 12:**
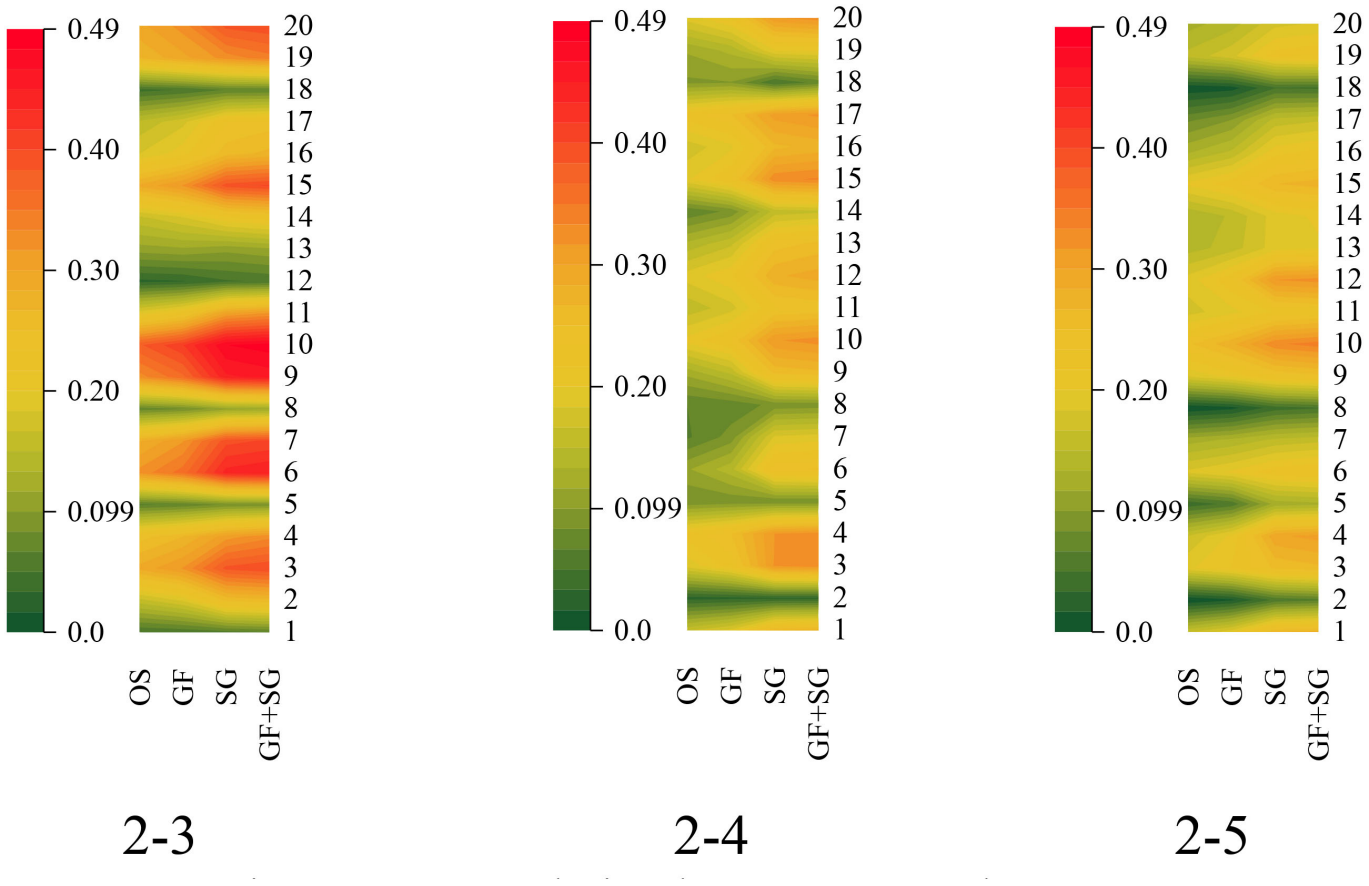
Correlations between the vegetation indices (VIs) and the leaf nitrogen content (LNC).

The calculated correlation coefficients between the VIs and cotton LNC are shown in **Figure 12**. The results indicated that VOGI, GRVI, RVI, MACI, RECI, GNDVI, NDVI, IPVI, mSR_705_, RENDVI, mND705, HNDVI, and SAVI were significantly correlated with LNC (*r* > 0.2). There was a substantial link between LNC and the ZMI, GRVI, RVI, MACI, GNDVI, NDVI, IPVI, MTCI, SIPI, RENDVI, mND_705_, HNDVI, and SAVI of 2-5 and the ZMI, GRVI, RVI, MACI, GNDVI, NDVI, IPVI, MTCI, SIPI, mSR705, and RENDV of 2-6. In contrast, R/G, GMI, and NPQI had no significant relationship with LNC. Nonetheless, the initial categorization findings revealed that R/G, GMI, and NPQI had a substantial relationship with LNC. All 20 VIs demonstrated a significant association with the cotton LNC in various picture pixel conditions. In general, the correlation coefficients between the indices and N in classifications 2 through 4 were higher than those in the other two categories. Spectral pre-processing greatly enhanced the correlation coefficients between the indices and LNC. The correlation between the OS and LNC was poor, but the correlation coefficient improved greatly after Gaussian filtering + SG smoothing, with GF&SG > SG > GF > OS offering the superior effect. GF&SG, SG, and GF all calculated indices with a superior link to LNC.

In conclusion, it can be seen that soil (1-2, 1-3, 1-7, 2-1, and 2-2) and shading (1-6) were effectively eliminated following the initial classification. After classifying the image pixels twice, the influence of soil on nitrogen monitoring was effectively minimized, and the link between the VIs and the cotton LNC was greatly improved.

The correlation coefficients of the indices with each other were greatly reduced after two classifications; however, the correlation coefficients of the indices with LNC were significantly increased. Consequently, this study will be an additional attempt to analyze the LNC based on a triple result of 2-3, 2-4, and 2-5 and with Pearson’s correlation coefficient to filter the sensitivity indices (**Table 6**).

**Table 6:** Sensitivity indices.

ID no.	Total	Sensitive Vegetation Index
2-3	14	VOG1, GRVI, RVI, MACI, RECI, GNDVI, NDVI, IPVI, mSR_705_, GNDVI, RENDVI, mND_705_, HNDVI, SAVI
2-4	15	ZMI, GRVI, RVI, MACI, RECI, GNDVI, NDVI, IPVI, MTCI, SIPI, GNDVI, RENDVI, mND_705_, HNDVI, SAVI
2-5	12	ZMI, GRVI, RVI, MACI, GNDVI, IPVI, MTCI, SIPI, mSR_705_, GNDVI, RENDVI, HNDVI

See **Table 2** for the definitions of the different indices.

### 3.4 Cotton nitrogen monitoring with machine learning

**Figure 13:**
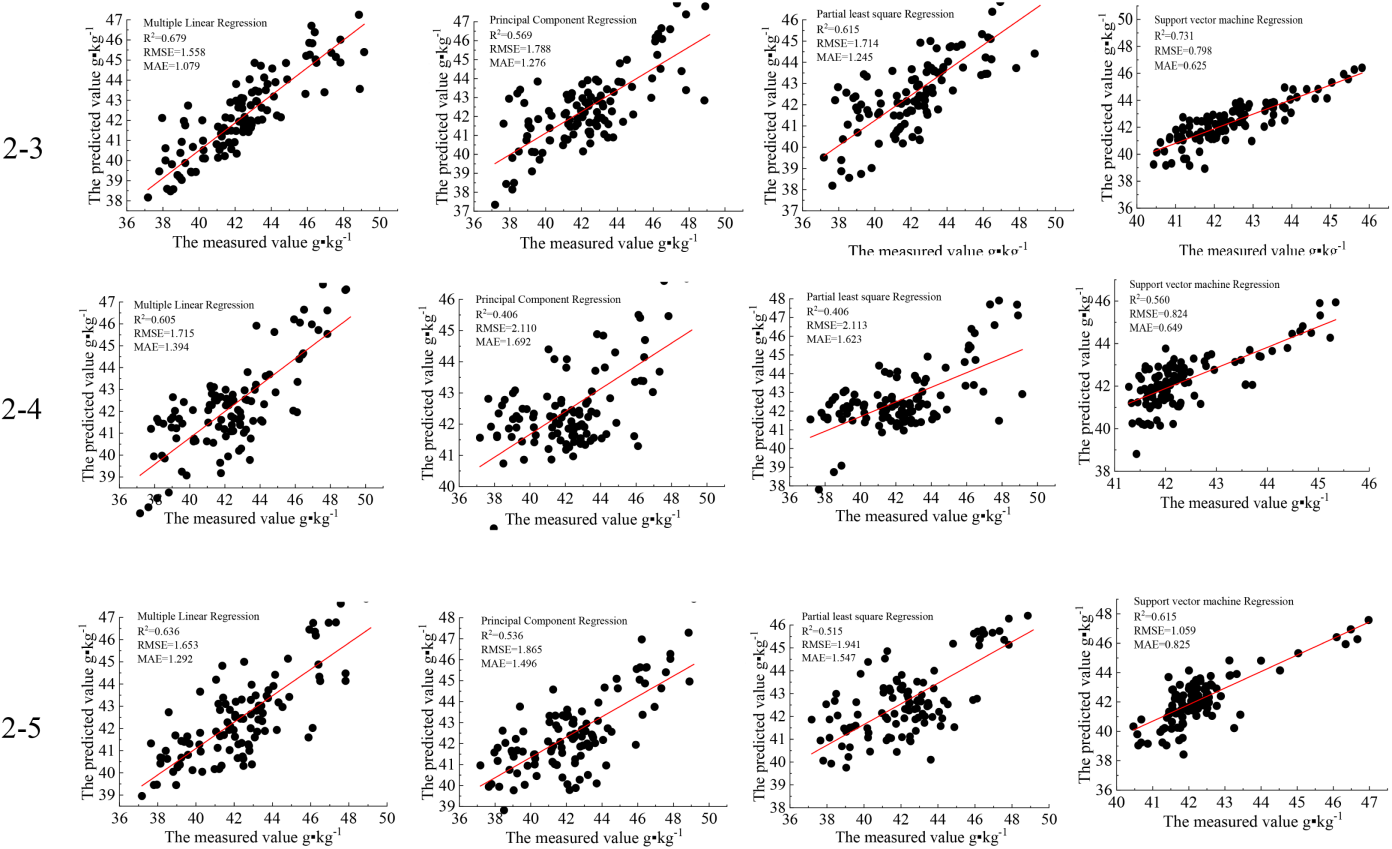
Results of cotton nitrogen monitoring.

Based on the VIs shown in Section 3.2, we used several machine learning methods to build a cotton nitrogen monitoring model. Because the same indices corresponding to 2-3, 2-4, and 2-5 had substantial differences, the indices in the three classifications were mixed at random to generate LNC models (**Figure 13**).

**Figure 14:**
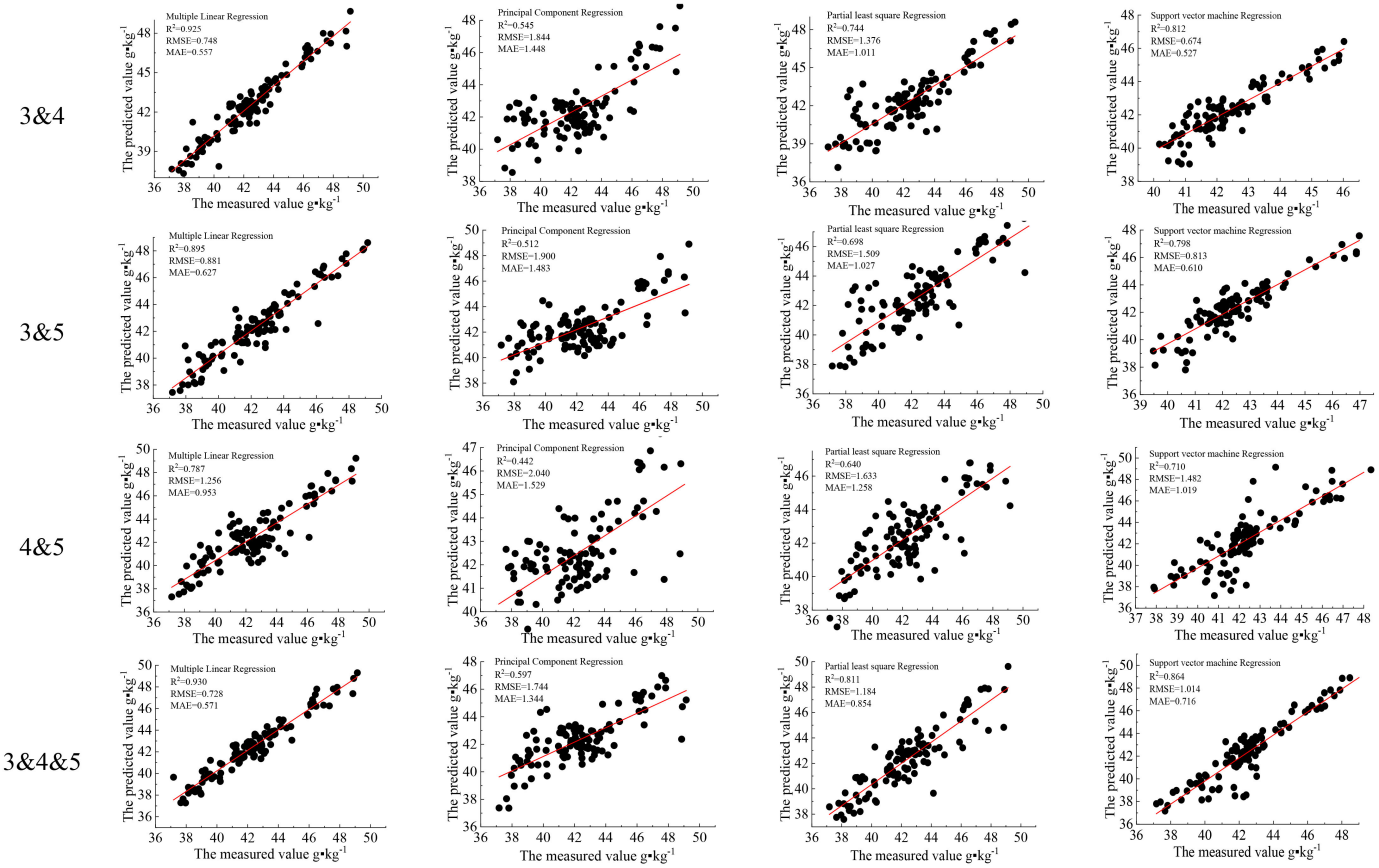
Monitoring of the leaf nitrogen content (LNC) of cotton after classification and combination.

It was determined that the accuracy of the model could be improved, particularly for the combination of VIs among the three categories, indicating that classed image pixels have a better performance in forecasting the LNC (**Figure 14**). This suggests that the categorized picture pixels are more capable of predicting N. It also demonstrates that the categorization presented in this research is an efficient method for N monitoring. Multiple linear regression showed the best prediction accuracy, followed by SVM.

## 4 Discussion and conclusion

### 4.1 Discussion

Currently, remote sensing images are popular for target detection in agriculture, but there are numerous challenges. Moharana and Dutta (2022) noted that one of the challenges of crop nutrient monitoring is the complexity of the farm environment, which interferes with spectral information due to the soil, leaf angle, and leaf shadow, among others. Sophisticated scenarios have been the focus of anti-interference research. The spectrometer consists mainly of an optoelectronic conversion, transmission, and processing system. Each module within the system produces noise at different levels, and the spectrum information of the true object is influenced by noises that are inevitable; hence, it is extremely essential to pre-process the spectrum (Jin et al., 2016). Wang et al. (2018) showed that the relationship between the UAV vegetation index and the soil water content could be improved after spectral pretreatment. In this study, after the spectra were pre-processed using Gaussian filtering, SG smoothing, and GF&SG, the correlations between the calculated spectral indices and the LNC increased gradually. Sun et al. (2017) performed pretreatment of the spectra for wheat flour gluten detection, and it was concluded that the model established after SG better matches the requirements of production detection than the OS. This is in agreement with the results of this study. When collecting spectral data for the monitoring of farmland information, spectral influence factors should be considered and the spectrum pre-processed purposefully.

Selected combinations of wavelength bands can be used to distinguish the plants from the soil background (Franz et al., 1991). In this study, GRVI, RVI, MACI, GMI, and MTCI were more susceptible than others to the disturbances of the surrounding environment, which may be due to the fact that these indices have common features that are made up of red edges, which is one of the distinctive characteristics of plants, whereas the four indices, VOG1, SIPI, NPQI, and HNDVI, were relatively stable than the others. Caturegli et al. (2015) pointed out that the size of the NDVI values are easily influenced by the surrounding environment. In this study, only 20 VIs proposed by previous authors were selected. The index found to be easily influenced by the surrounding environment was the ratio index. The normalized VIs had a more stable effect. There are various VIs that are prone to saturation, and their role in crop responsiveness to disturbance is unclear. Therefore, the use of more vegetation indices with clearer functions can be attempted at a later time. An index that can significantly distinguish shading and vegetation is important for agricultural information monitoring. In addition to VIs, disturbance rejection has been studied by other researchers. For instance, Woebbecke et al. (1995) used reflectance and field-of-view analysis to create a straightforward optical plant sensor that can distinguish plants from their surroundings, including soil and plant garbage. It is suggested that an RGB master system-based color scheme is used to distinguish plants from their natural environment.

There are currently many studies related to shadow detection. The shadow regions in a hyperspectral image have extremely low values. Therefore, these regions are either directly deleted or ignored in target detection or classification (Liu et al., 2020). Consequently, there are some studies focused on improving the reflectivity of the shadow regions; however, it remains difficult to determine the actual substances contained in these shadow regions. M-Desa et al. (2022) proposed that shadow recognition in digital images is an essential step in pre-processing for computer vision as the shadows in images can hide the features of the target objects in detail. However, there are only a few studies on crop shadow detection. Therefore, it is necessary to focus on the impact of shadows on the monitoring of farmland information in future studies. For example, Imai et al. (2019) used a UAV to obtain hyperspectral images of orange plantations in multiple wavelengths. Wavelength information near blue light and long-wave NIR has been proposed to be able to identify farmland and shadows well. Li et al. (2022) divided the apple canopy into shade and canopy using the threshold method. However, the canopy shading of orange and apple is not as complex as that of agricultural field shading. Hence, efficient distinction between the agricultural shadow and the plant cover will be one of the issues that should be resolved in agricultural research. In this study, the image pixels were classified into shadows based on the greenness of plant growth, while the soil and a number of useless shadows were removed. Cotton is a dynamic crop, which makes shading more complex; therefore, the canopy spectra of cotton are easily impacted by shadow. This study provides a method for classifying image pixels based on the intensity of green light. On the one hand, this is due to the fact that chlorophyll has a significant association with nitrogen (Barker and Sawyer, 2010; Schmidt et al., 2011). Traditional nitrogen monitoring involves assessment of the quantity of nitrogen fertilizer based on the leaf color, mostly due to the intensity of the leaf color being correlated with nitrogen (Bacsa et al., 2019). Chlorophyll is directly proportional to the intensity of the leaf color in plants. On the other hand, green light, the greatest distinguishing element of green plants, can be used to clearly differentiate vegetation from other backdrops. In this study, the vegetation image pixels were classified using the green light, and the image pixels were classified twice based on the level of vegetation greenness. The correlations among the VIs were obviously decreased after the second classification, while the correlations between the indices and nitrogen were improved. This indicates that the spectral reflectance and vegetation indices of the image pixels each have great differences and that the classification can effectively eliminate the interference of soil and shadow. In the 1980s, Guyer et al. discovered that the intensity of the pixels in the plant images was higher than that of the soil pixels by shining visible light on the plants. This indicates that grading the intensity of green light is significantly effective for soil and shadow rejection. The classification should be further improved in the future, so that shadows can be distinguished more clearly.

In this study, several monitoring models were developed based on the classification results using common methods. It was found that the results showed good performance. Among them, MLR and random forest regression had the most positive results. Manfreda et al. (2018) proposed that the random forest algorithm combined with VIs has superior performance in predicting the canopy nitrogen in citrus. Moreover, since the indices of each classification reached a significant relationship with nitrogen after two classifications, in this study, the indices of three classifications were combined for modeling. The contributions of each classification to nitrogen should be considered, and weights should be assigned to each of the indices in a future study, which might reach better results.

### 4.2 Conclusion

In this study, we employed a UAV hyperspectrum to acquire hyperspectral images, classified the image pixels twice to green light, and then excluded the unrelated image pixels to capture the relevant image pixels. Vegetation indicators were retrieved using multiple pretreatment methods to construct an LNC monitoring model. The results demonstrated that the classification proposed in this study can successfully eliminate the mixing spectra from the shadows of cotton leaves and soil, as well as improve the relationship between LNC and VI. In addition, GF&SG served as a de-interference mechanism for the UAV spectra. The LNC of cotton could be well known when combined with various machine learning techniques. Consequently, while monitoring nitrogen using UAV hyperspectral images, it is crucial to consider extracting the pertinent properties of the imaging spectra, such that the spectrum captured is more indicative of the cotton leaf spectrum reflection and nitrogen monitoring accuracy.

## Data Availability

The original contributions presented in the study are included in the article/**Supplementary Material**. Further inquiries can be directed to the corresponding authors.

## Supplementary material

Supplementary Figure 1 DJI M600.

Supplementary Figure 2 Sensor.

Supplementary Figure 3 Region of interest (ROI). The first ROI, 900 image pixels, excluding the soil between the cotton rows, was rejected in **Figure 2**.
