# Supplementary figures and images for: Reducing soil and leaf shadow interference in UAV imagery for cotton nitrogen monitoring
Source: Front Plant Sci. 2024 Aug 16;15:1380306. doi: 10.3389/fpls.2024.1380306 (PMC11362076; doi:10.3389/fpls.2024.1380306)

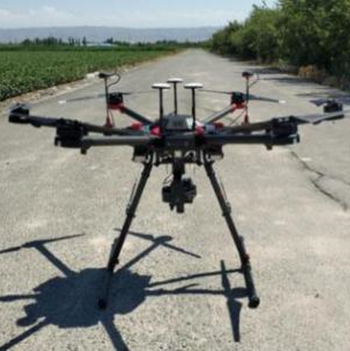

Supplement: Supplementary Figure 1 — DJI M600. [file Image1.tif]

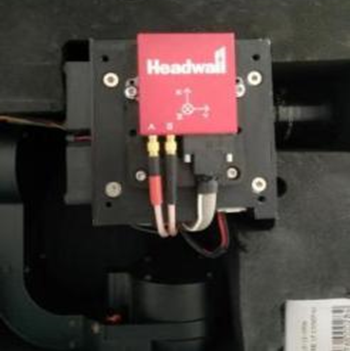

Supplement: Supplementary Figure 2 — Sensor. [file Image2.tif]

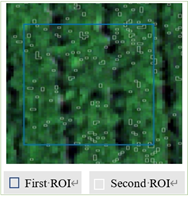

Supplement: Supplementary Figure 3 — Region of interest (ROI). The first ROI, 900 image pixels, excluding the soil between the cotton rows, was rejected in Figure 2 . [file Image3.tif]
